# Supplementary material for: A critical analysis of the What3Words geocoding algorithm
Source: PLoS One. 2023 Oct 25;18(10):e0292491. doi: 10.1371/journal.pone.0292491 (PMC10599581; doi:10.1371/journal.pone.0292491)
Supplement: S1 File — (PDF) [file pone.0292491.s001.pdf]

Appendix A: Sensitivity Analysis for $p_3(c_\Pi > 3)$

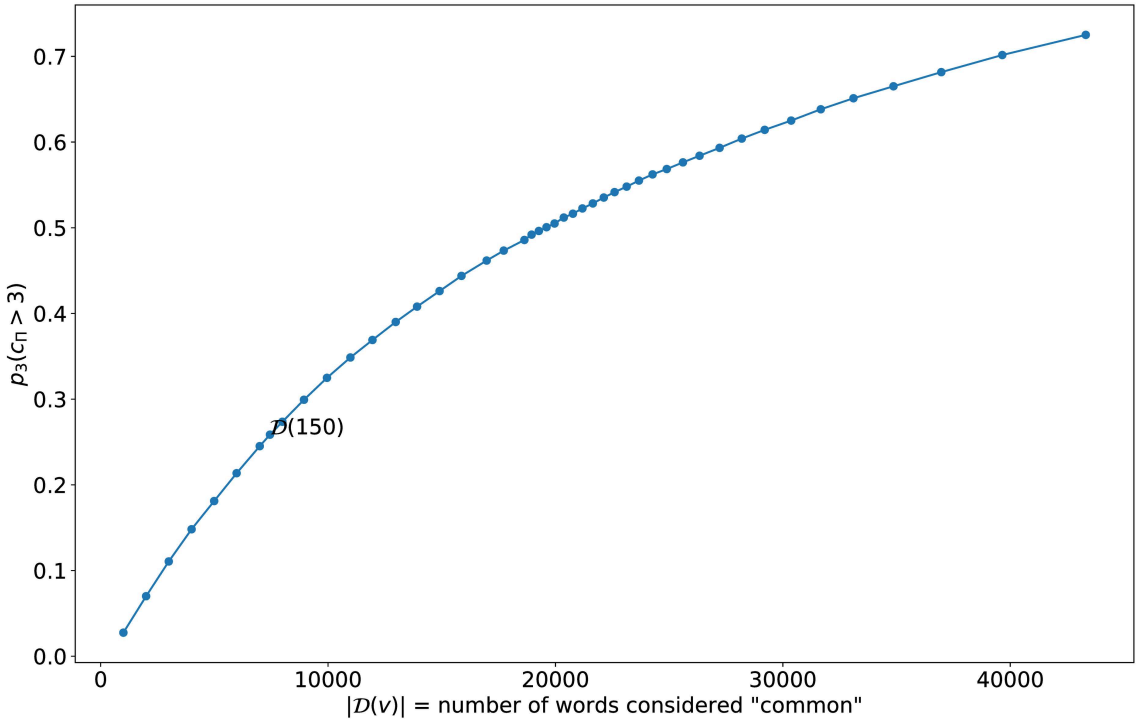

Fig 3. Sensitivity analysis showing the dependence of $p_3(c_\Pi > 3)$, the fraction of addresses with more than 3 confusions, on the size of the 'common' set, $|\mathcal{D}(v)|$.

While the conclusions remain the same, the exact values reported above vary depending on our cutoff for defining a "common" word. To re-iterate, I only permit words in W3W addresses to be confused with common words, since it seems unlikely someone would confuse a common word like, say, 'rust', with an unusual one, like 'roust'. Common words are defined as words in the set $\mathcal{D}(150)$, which are the approximately 7,500 most common words in the corpus of [1]. Fig 3 shows the dependence of the key quantity $p_3(c_\Pi > 3)$, the fraction of addresses with more than 3 confusions, as the size of the 'common' set, $|\mathcal{D}(v)|$, is varied. The choice of $\mathcal{D}(150)$ is relatively conservative, however, even more restrictive definitions of 'common', say $|\mathcal{D}(600)| \simeq 3000$, still give a significant number of addresses with more than three confusions, over 10% in this case. A wider definition of 'common' obviously makes the situation considerably worse.

Appendix B: Expected Number of Confusions in an Area

Let A be the event that there is a circle of radius r containing $a(r)$ addresses with no confusions. $a(r) \simeq \pi(r/d)^2$ where $d = 3m$ is the grid size. $\hat{A}$ is the event we have at least 1 confusion in the area and $P(\hat{A}) = 1 - P(A)$. Finding $P(\hat{A})$ is equivalent to the so-called Birthday Problem [2]. Number the $a(r)$ addresses 1 to $a(r)$. The event that no addresses are similar is the same as the event that address 2 is not similar to address 1, *and* that address 3 is not similar to either 1 or 2, and so on. Let these events be called Event 2, Event 3 etc.

There are $T \sim 5.7 \times 10^{13}$ W3W addresses in total. Assume address i has c_i confusions. So that we can solve analytically, make the simplification that there are exactly c similar addresses for every address. The probability of Event 2 is then $(T - c)/T$, as address 2 may have any address not similar to address 1. The probability of Event 3 given that Event 2 occurred is then $(T - 2c)/T$, as address 3 may be any addresses not confusable with 1 and 2. The pattern continues until Event $a(r)$, which has probability $(T - (a(r) - 1)c)/T$. $P(A)$ is equal to the product of these individual probabilities:

$$P(A) = \frac{T}{T}\frac{T-c}{T}\frac{T-2c}{T}\dots\frac{(T-(a(r)-1)c)}{T} = \frac{c}{T^{a(r)}}\frac{\frac{T}{c}!}{(\frac{T}{c}-a(r))!}$$

and $P(\hat{A}) = 1 - P(A)$ gives us our target probability.

This can be made more tractable by making some approximations, because T is very big

$$\left(1 - \frac{kc}{T}\right) \simeq \exp(-\frac{kc}{T})$$

and

$$\begin{aligned} P(A) &= \frac{T}{T}\frac{T-c}{T}\frac{T-2c}{T}\dots\frac{(T-(a(r)-1)c)}{T} \\ &\simeq \exp\left(-\frac{c}{T}(1+2+\dots a(r)-1)\right) \\ &\simeq \exp\left(-\frac{ca(r)^2}{2T}\right) \end{aligned}$$

and so

$$P(\hat{A}) \simeq 1 - \exp\left(-\frac{ca(r)^2}{2T}\right)$$

References

1. Brysbaert M, New B. Moving beyond Kučera and Francis: A critical evaluation of current word frequency norms and the introduction of a new and improved word frequency measure for American English. Behavior research methods. 2009;41(4):977–990.
2. Knuth DE. The art of computer programming, volume 3: Sorting and searching. vol. 3. Pearson Education India; 1973.
