# Supplementary material for: A critical analysis of the What3Words geocoding algorithm
Source: PLoS One. 2023 Oct 25;18(10):e0292491. doi: 10.1371/journal.pone.0292491 (PMC10599581; doi:10.1371/journal.pone.0292491)
Supplement: Attachment — Submitted filename: Response to Reviewers.pdf [file pone.0292491.s002.pdf]

Response to Editor:

1. Please ensure that your manuscript meets PLOS ONE's style requirements, including those for file naming. The PLOS ONE style templates can be found at

https://journals.plos.org/plosone/s/file?id=wjVg/PLOSOne_formatting_sample_main_body.pdf and

https://journals.plos.org/plosone/s/file?id=ba62/PLOSOne_formatting_sample_title_authors_affiliations.pdf

In the reuploaded version I have used the PLOS LaTeX template and followed the guidelines to the best of my knowledge

2. Please note that PLOS ONE has specific guidelines on code sharing for submissions in which author-generated code underpins the findings in the manuscript. In these cases, all author-generated code must be made available without restrictions upon publication of the work. Please review our guidelines at https://journals.plos.org/plosone/s/materials-and-software-sharing#loc-sharing-code and ensure that your code is shared in a way that follows best practice and facilitates reproducibility and reuse.

The code has been given a DOI which is referenced in the paper.

3. We note that you have stated that you will provide repository information for your data at acceptance. Should your manuscript be accepted for publication, we will hold it until you provide the relevant accession numbers or DOIs necessary to access your data. If you wish to make changes to your Data Availability statement, please describe these changes in your cover letter and we will update your Data Availability statement to reflect the information you provide.

There is no data source unique to this project, all data is obtained from other published works referenced in the text.

4. Please amend either the abstract on the online submission form (via Edit Submission) or the abstract in the manuscript so that they are identical.

I have done so.

5. We note that you have referenced (Wood N. Autocorrect awareness: Categorizing autocorrect changes and measuring authorial perceptions) which has currently not yet been accepted for publication. Please remove this from your References and amend this to state in the body of your manuscript: (ie "Bewick et al. [Unpublished]") as detailed online in our guide for authors

http://journals.plos.org/plosone/s/submission-guidelines#loc-reference-style

The work in question is reference number 13 and appears in the bibliography as [Unpublished master's thesis], given the use of this work to simply provide an interesting example of a potential substitution made by an autocorrect algorithm, I think this is an appropriate document to reference.

6. Please review your reference list to ensure that it is complete and correct. If you have cited papers that have been retracted, please include the rationale for doing so in the manuscript text, or remove these references and replace them with relevant current references. Any changes to the reference list should be mentioned in the rebuttal letter that accompanies your revised manuscript. If you need to cite a retracted article, indicate the article's retracted status in the References list and also include a citation and full reference for the retraction notice.

I believe the reference list is complete. I do not believe I have referenced any retrated work.

Response to Reviewer 1:

Reviewer #1: Overall:
This is generally a well-written and well-organised paper that assesses the reliability of the What3Words geocoding application. The paper applies a convincing methodology to explore the potential for confusing different addresses – despite the W3W algorithm not being open-source. I have read the paper with great pleasure, and I only have some minor issues which I think should be addressed before publication.

I thank the reviewer for their kind assessment of the work

Comments:
- Section 1 is a little confusing as it is explained that W3W uses a closed-source algorithm (p. 1) and when the aim of the paper is subsequently introduced it does not become immediately clear how it is possible to analyse the algorithm.

Apologies for the confusion, this has been changed to closed-source code.

- The paper quite heavily relies on the idea that because W3W is widely adopted by emergency services, it needs to minimise any source of confusion (Section 1). This is supported by a reference that 85% of emergency services in the UK use W3W as well as that there is a website devoted to W3W issues. However, the "85%" is a claim made by W3W themselves (without a reference) and the dedicated website has not been updated in over a year – it also only received a handful of updates in 2022. The paper would benefit from some further justification or further statistics, where possible, on the general adoption of the W3W application (i.e. in Section 1).

It is quite hard to get reliable usage statistics on W3W usage from the company, beyond self-reported numbers like the 85% quoted above. I have added the following which provides some further evidence for use of W3W in emergency applications and reports the 85% claim of W3W more sceptically.

W3W report various applications of their system in logistics, taxi services and navigation [3]. W3W also share numerous instances of use of the app by emergency services, notably the claim that W3W is used by 85% of emergency services in the UK [4]. While claims from W3W themselves may be treated with some scepticism, it is the case that major organisations like car breakdown service the AA [5] and the London ambulance service [6] encourage and give advice on using W3W for reporting

locations. Other agencies, such as Mountain Rescue England and Wales, advise using it only in conjunction with other location methods [7].

It is for applications in emergency situations that W3W has been the target of robust criticism in the popular literature for, among other issues, its closed-source code [8] and the potential for confusion between addresses [9-11]. A website devoted to issues arising from use of W3W has been established at `https://w3w.me.ss/`. If W3W is widely adopted by emergency services it must be subject to rigorous evaluation.

- The description of the W3W application (Section 2) is very technical, which is fine, but it would benefit from a bit more of a general explanation how a user would interact with the application, for instance, in case of an emergency. I think a simple map showing part of the grid with associated words would be beneficial before moving on to the details of the algorithm.

The w3w app itself is freely available and almost self explanatory so I think a link might be more useful here. I have added the following to the start of the Section "The What3Words Algorithm"

A user of the W3W service simply interacts with the mobile application or website `https://what3words.com/` either by entering a W3W address to get its location on a map (geocoding) or tapping the locate button to obtain their W3W address (reverse geocoding). The details of transforming between geodesic coordinates and W3W addresses are hidden from the user and the assignment of words to locations is fixed. It is the process of assigning word triples to locations, as described in the What3Words patent [14], that will concern us here.

- The description of how the word list is created (Section 3.3) could be improved in clarity. Why is 'D(150)' selected rather than, for instance, 'D(1000)' or '(D200)'. Perhaps some sensitivity analysis would be helpful as this seems to have quite an impact on the size of the confusion set?

To improve the description I have provided a worked example for a particular word:

For example take w = 'clause'. To make $C_t(w)$ generate mutations of w using the typing error modes described in [19].

1. Transposition, generating e.g. 'cluase'

2. Adding an extra letter e.g. 'clauses'

3. Deleting a letter e.g. 'cause'

4. Swapping a letter e.g. 'claude'

There is only one homonym of 'clause' in the word list so $C_h(w)$ ={claws}. The union of $C_t(w)$ and $C_h(w)$ is then intersected with D(150) which removes invalid words (like 'cluase') and rare words. The final result is C(clause) ={clause, cause, claws}

The threshold of 150 was chosen because it returns roughly only words which would be within the first 3 `bands' of w3w cells - these would be populated places where people are likely to use w3w. The words chosen are among the top ~7500 in the English language i.e. common enough that people (and spell checkers) would most likely correct a potential misspelling or mishearing of a similar word to a word in this set.

I think the choice of 150 is justified by these considerations, and indeed this number is quite small and therefore favourable for w3w in terms of allowing a smaller number of potential confusions per word. However the reviewer is correct that results are sensitive to this threshold so I have added an appendix demonstrating the sensitivity of the exact values reported on the choice of D(150). See Appendix A in the updated draft which shows the qualitative conclusions i.e. that there are many addresses with more than 3 confusions, don't change and indeed shows that D(150) is a fairly conservative (i.e. good for w3w) estimate and things are much worse for larger values.

Minor comments:
- Table 1: 'tree' and 'three' are homophones my accent but not in others. This sentence seems to be incomplete.

Changed to: `tree' and `three' are homophones in my accent but not in other accents

- "However in practice many can be found and, as mentioned in Table 1, homophony is dependent on accent and it is unlikely that all homophones across all regional accents, even in the UK, can be removed." (p.4). Is there any way to include a (rough) estimate on how many homophones are likely to be included in the W3W word list?

Since w3w say that they prune the word list to remove words which have homophones [22] this is quite difficult, since a number of "obvious" ones like "witch" and "which" don't seem to be present but many can be found e.g. "arose" and "arrows" it is hard to know the true number without seeing the word list. The number of homophones in my word list may not be comparable. Likewise there is also the problem mentioned above, regional dialect variation means homophones are not constant across space. I have added the following

There are 1699 words in D that have at least one homonym also in the dictionary. Many of these are unusual words e.g. 'sitz'. Restricting the set to only count words in D that are homophonous with common words (D(150) see next section for the definition) gives 650 words with common homophones e.g. 'sense' and 'cents'. The word list used by W3W is pruned in some unknown way so these numbers are only estimates. However, anecdotal evidence e.g. [10] and experimentation with the app suggests it is not hard to find addresses in W3W containing a word with a homophone, for example 'arrows.midst.sense' is a valid address in Queensland, Australia.

- "…only lower case words which appear at least 3 times in the corpus of [19] are kept." (p.5). It is not clear to me how words can appear multiple times in a frequency-based word list?

The word list is derived by counting words in a large collection of documents (the corpus). So each word has a corresponding count for the number of times it appears, very common words have high counts, unusual words have low counts. For the particular corpus used by

[19] (television subtitles) requiring a count greater or equal to 3 corresponds to words like ‘replayed’ which are likely to be understood by all fluent English speakers while giving a word list of comparable size to the real word list of w3w.

- “Generate the set of all strings that could be formed by making the typing errors listed above to make Ct(w).” (p.5). How exactly?

Hopefully the ‘clause’ example has clarified this.

- Figure 2b is not really clear and I find it difficult to interpret. Perhaps this would work better for a smaller area?

I thank the reviewer for bringing this to my attention. In the original version I did not include the additional cell to clearly show the effect discussed in the preceding text. An additional cell has now been added showing the $\Delta m=1$ problem, as well as a figure focussing on just these confusions. I have also expanded the discussion. See the new Figure 2, its caption and the discussion reproduced below:

To see this I simulated four W3W cells with $Y = 3396$, $X = 4316$ and $q = 0$. In the first cell I found 685 confusable pairs. Simulating four neighbouring cells with $q = i \times 1, 485, 706$ for $i \in \{0, 1, 2, 3\}$ results in a roughly twenty fold increase in the number of confusions to 11,173. Figure 2 (a) shows the distribution of distances between confusable pairs across 4 band-0 cells, covering an area of roughly 14.5km × 4.5km. Many are quite close together. Fig 2 (b) shows the locations of these confusable pairs (size exaggerated for visibility), coloured according to if there are $Ne = 0$, 1 or 2 words shared and in identical position between confusable addresses. As seen in (a) and highlighted in (c) and (d) there is a significant peak in confusable pairs separated by around 10km, which corresponds to $\Delta m = 1$.

As well as this peak, there are a large number of other confusable addresses within these 4 cells. $\Delta m = 1$ is the most extreme case, but any Δn giving a small Δm will reduce the effective address space. One should also consider Δn values that connect confusable rather than identical words, for example runs of addresses like await.v.w and awaits.v′ .w′ . As many potential word confusions exist, there are many ways to make these problematic pairs, and this will be the case for any constants a and b. Thus the effective address space is much less than L^3 or even K^3 and Eq 6 grossly underestimates the number of confusing address pairs.

Response to Reviewer 2:

Reviewer #2: PONE-D-23-18725
Studying the What3Words Geocoding System

Review

The paper presents an initiative to assess important characteristics of the What3Words (W3W) geocoding system, in face of anecdotical evidence of confusion involving 3-word addresses, composed of common English language words, provided by the system.

Since W3W is not open software, nor does it open its reference data (specially the list of words used to compose the 3-word addresses), a direct investigation would require executing a large number of accesses, in order to gather actual W3W data. These accesses have a cost, so the author instead devised a strategy to simulate the W3W algorithm and list of words, in order to perform the intended analyses. Furthermore, as the terms of use do not allow storing addresses along with their coordinates, even if large numbers of accesses were free, an external analyst would have trouble in devising a fair assessment method.

I thank the reviewer for their assessment of the work.

As a result, readers should keep in mind that the analyses presented in the paper do not actually refer to W3W as an online service, but to a simulation of W3W based on what is known about its basic working logic, algorithms, and word selection strategy. The title and many statements along the text should reflect this fact. The paper's title, for instance, could be changed to something like "A simulation and critical analysis of the What3Words geocoding system".

I agree with the reviewer here. I have changed the title to "A Critical Analysis of the What3Words Geocoding Algorithm", since that is what's done in the paper!

In the path towards creating a W3W simulation, the author makes a number of assumptions, especially on the constitution of the list of words that are indexed by the method. In the paper, a text corpus is used to obtain a list of English words along with their frequencies, from which words with less than 4 characters are discarded and the most common remaining ones are selected. The actual selection process in W3W is unknown, but (again) anecdotical evidence is used to show a number of issues, such as the use of plurals, similar-sounding groups of words, spelling variations and more.

One can fairly easily find words and their plurals on the w3w app by typing almost any address. As the reviewer correctly states, since the exact word list is hidden, the exact number of such cases in practice is unknown.

The paper shows the occurrence of the types of confusion that might happen in actual W3W addresses, and demonstrates that they occur quite frequently in the simulated setting, with the selection of words devised for this study (not the actual W3W list of words). Given the size of the corpus, the number of words in the list, and the publicly known characteristics of W3W, the simulation seems fair, but its conclusions are more directly applicable to the simulation, rather than to the actual service. Conclusions, therefore, are indirect – and, of course, there would be no other way to draw more precise conclusions, other than by having access to the actual W3W code and data, or maybe a large number of queries to the system.

I fully agree with the reviewer on these points

The indirect conclusions are not a problem per se, but naturally there remain some additional doubts regarding their applicability to the actual service. The qualitative assessments presented as conclusions could be strengthened if verified using the actual service. This, of course, would require acquiring a license to use W3W for some time period. I list below a few possible strategies for doing so:

1. Randomly select W3W addresses, and verify their vicinity in search for nearby confusing addresses up to some maximum distance. Verify if the results fall within the probability determined in the simulation.
2. Generate a number of confusing addresses from the simulation data, and check for the existence of the confusing versions in actual W3W; then verify their distance.
Example: small.dark.places (mentioned as an example in the paper) exists in Edinburgh, UK; dark.small.places exists in Illinois, USA. Large distance, not a problem.
3. Pick a pair of words for the first two positions, and then vary the third using words from the simulation list. Analyze the spatial distribution of the valid results.

I agree that it would be ideal to study the actual w3w code, rather than the algorithm as described in the patent. I have considered options similar to the ones suggested above, however since no funding is available to me to purchase API access and I am required to abide by the app TOS - the free API only allows 100 requests per month https://accounts.what3words.com/select-plan (Accessed 16/08/23), which would not be sufficient for the proposed analyses.

I note that independent security researchers have performed some reverse engineering on w3w and do find the described artefacts in practice, see references 9 and 10 from the paper. See also reference 11 (added) where rescue workers report numerous incorrect w3w addresses they have received.

I also note something interesting about the example used in 2: s*mall.dark.places* is actually in Edinburgh, Indiana, close to Indianapolis, a neighbouring city of Chicago, where *dark.small.places* is located. These are similar addresses in neighbouring cities, so could reasonably be confused. Furthermore, if I type *dark.small.places* into w3w (https://what3words.com/dark.small.places) *small.dark.places* does not appear on the list of suggested locations - an error of the first type described in the paper.

Given, however, the stated limitations of this study, the simulation approach is adequate and the paper effectively shows weak points and potential problems with the W3W algorithm and related data. I suggest revising the phrasing on some claims, assessments and estimations, in order to make it clear the extent to which the simulation is expected to accurately replace the behavior of the actual system.

I agree and have edited the manuscript in line with the suggestions below.

I also would like to point out the importance of making these evaluations widely known since, while agreeing with the author's admonitions against the use of W3W by voice over noisy channels, I can clearly see that W3W can be improved based on the systematic characterization of its potential problems. Various alternatives come to mind, but I'll refrain

from mentioning them here, as solutions are not within the purview neither of the paper, nor of the review.

I also agree that a w3w-like system could be developed without these limitations, however as suggested, this is out of scope for this paper.

Localized issues, questions, suggestions:

1. Abstract: … for being less reliable THAN claimed (typo)

Fixed

2. Section 4: W3W's claim on the overwhelming proportion of similar-sounding word combinations being far apart cannot actually be verified in the simulation, so I suggest rephrasing the second sentence. Also, your observation that the autocorrection feature "seems to balance address similarity with prior information about places" should be better explained. Did you verify that suggestions are of addresses near the user?

I changed the sentence to: We will study if the W3W algorithm places similar addresses far apart in the next section

The AutoSuggest is another closed-source part of the w3w algorithm. I am not aware of any public description of this algorithm beyond a description of it given by w3w in ref [26]. I have added the following
Correcting an incorrect address relies on the AutoSuggest algorithm implemented in the W3W application [26] which displays 3 suggested addresses based on the user's input. The algorithm used is closed source and the description in [26] only mentions 'different factors' accounting for which addresses are suggested. However they do state: *In certain situations, like an e-commerce checkout, we increase the likelihood of an accurate match by clipping the area of results to the shipping country.* From these statements, personal experimentation and information given by the help page [27], AutoSuggest seems to balance address similarity with prior information about places and seems to be most likely to make suggestions of populated places near the user.

I note that the details of this algorithm are not really crucial to the results presented, only the fact that it returns at most 3 suggestions.

3. The lost hiker scenario could possibly be resolved if the hiker transmits two W3W addresses, e.g. for neighboring cells. Chances of a mistake in both of them at the same time, directing responders to a different nearby place with the two distortions close to each other should be very low. Of course, transmitting too much defeats the purpose of using W3W in the first place: it then could be better to spell out each digit of the lat and lon coordinates, or to spell out each letter of each of the three words using ICAO (alpha-bravo--charlie-)…

I have added the following

W3W ambiguity could be resolved by transmitting multiple addresses or spelling out the words, but then the main advantage of W3W, simplicity, is lost. Also in some cases e.g. a lost, injured hiker, this might not be possible.

4. Page 16, first paragraph: "I generated three W3W cells…" -> "I simulated the composition of three W3W cells using the word list from Section 3."

Changed `generated' to `simulated'

5. Conclusions: "Most W3W addresses have one or more word triples with which they could be confused" -> Most of the simulated W3W addresses tend to have one or more triples… or Simulation results indicate that addresses created using the W3W method have one or more…

Changed to: Most of the simulated W3W addresses have one or more word triples with which they could be confused.

6. Conclusions: "is far in excess of" -> simulation indicates that the likelihood of…

Changed to:
The simulations performed suggest that the likelihood of nearby confusable pairs is far in excess of the very low (1 in 2.5 million [29]) likelihood quoted by W3W.

7. Page 17, "Departement"-> Department (? typo)

Fixed

8. Conclusions: "…this work has shown that many confusable W3W addresses exist…" -> many confusable W3W addresses are likely to exist.

Changed

9. Conclusions: "This work has tested some of the claims that W3W makes about their algorithm. These claims are not borne out under scrutiny which suggests that W3W should not be adopted as critical infrastructure without a thorough evaluation against a number of competing alternatives." I totally agree with these statements.

I thank the reviewer for their careful reading of the manuscript.
